# Imported and autochthonous malaria in West Saudi Arabia: results from a reference hospital

**DOI:** 10.1186/s12936-018-2438-7

**Published:** 2018-08-07

**Authors:** Rasha Hassan Soliman, Patricia Garcia-Aranda, Sherine Mohamed Elzagawy, Boshra El-Sayed Hussein, Wael Wahid Mayah, Alexandra Martin Ramirez, Thuy-Huong Ta-Tang, José Miguel Rubio

**Affiliations:** 1 Microbiology Department, Faculty of Medicine, Taif University, Al Hawiyah, Taif, Kingdom of Saudi Arabia; 2 Parasitology Department, Faculty of Medicine, Suez Canal University, Ismailia, Egypt; 3 Malaria and Emerging Parasitic Diseases Laboratory, National Microbiology Centre, Instituto de Salud Carlos III, Madrid, Spain; 4 Princes Nourah Bint Abdulrahman University, Riyadh, Kingdom of Saudi Arabia; 5 Department of Tropical Medicine and Infectious Diseases, Faculty of Medicine, Tanta University, Tanta, Egypt; 6 King Faisal Medical Complex, Taif, Kingdom of Saudi Arabia; 7 Faculty of Dentisary, King Abdulaziz University, Jeddah, Kingdom of Saudi Arabia

**Keywords:** *Plasmodium falciparum*, Drug resistance, Antifolate drugs, Saudi Arabia, Indigenous cases

## Abstract

**Background:**

The Kingdom of Saudi Arabia is seeking malaria eradication. Malaria transmission has been very low over the last few years. Discovered cases of *Plasmodium falciparum* infection are assigned a treatment protocol of artemisinin-based combination therapy, which consists of artesunate in addition to sulfadoxine-pyrimethamine rather than the traditional chloroquine, which has high resistance rates worldwide. This study aims to investigate the presence of different gene mutations concerning anti-malarial drug resistance (*pfdhfr, pfdhps, pfmdr1, pfcrt, pfcytb, pfketch13*) to identify whether drug-resistant alleles are present in this area of the Kingdom and whether the country’s treatment protocol is still suitable for *Plasmodium* bearing a resistance mutation.

**Methods:**

Blood samples were collected from patients suffering from symptoms suggesting malaria coming to King Faisal Hospital, Taif, from February to August 2016. Diagnosis was performed by Giemsa-stained thin and thick blood films, rapid diagnostic test and PCR. Positive *P. falciparum* samples were further subjected to series of PCR amplification reactions targeting genes related with drug resistance (*pfdhfr, pfdhps, pfmdr1, pfcrt, pfcytb*, *pfketch13*).

**Results:**

Twenty-six cases were positives, 13 infected with *P. falciparum*, of those, 4 cases were autochthonous, and 13 with *Plasmodium vivax*. The results of the gene mutation detection confirmed that there was no mutation related to resistance to artemisinin or atovaquone, on the other hand chloroquine resistance alleles were detected in 31% of samples. Moreover, point mutations in the *pfdhfr* and *pfdhps* genes, related resistance to antifolate drugs, were detected in all characterized samples.

**Conclusions:**

Haplotypes of *P. falciparum* in the western region of the Kingdom of Saudi Arabia exhibit high resistance against antifolate drugs. These results should be extensively discussed when planning to modify anti-malarial drug protocols in the future.

## Background

Malaria is considered one of the main ongoing causes of morbidity and mortality globally, affecting vulnerable groups, such as pregnant women and children [1]. This major health hazard is equally encountered in countries with regular transmission as well as those where transmission has been largely controlled or eliminated [2].

The Arabian Peninsula stretches out at the fringes of malaria endemicity where disease transmission has undergone different stages of reduction as a consequence of continuous vector control efforts. In Saudi Arabia, the biggest country in the gulf area with more of 32 million inhabitants, malaria was a noteworthy health issue in the middle of the last Century with frequent transmission all over the country, with 48,000 people living in foci areas [1]. Nevertheless, elimination of the disease in many regions was established in 1957 after the introduction of a systematic control programme based on dynamic case detection, enhanced surveillance and vector control, however, it remains sporadic and resilient in some areas [3–7]. Saudi Arabia is included in the E-2020 initiative by the World Health Organization (WHO), given a target of zero indigenous cases for 2020, but in the last published data, the country shows a rise in cases passing from 83 in 2015 to 272 in 2016 [1]. In addition, there are around 5000 imported malaria cases. *Plasmodium falciparum* counts for most of the cases of indigenous malaria, 270 in 2016, with sporadic cases of *Plasmodium vivax* (two in the last WHO report [1]).

According to malaria endemicity, Saudi Arabia can be partitioned into four topographical categories: (i) non-malarious areas lying in the centre of the country, where *Anopheles sergentii* is detected in low density and occasional imported cases may be reported; (ii) northern and eastern parts of the country where effective control measures resulted in termination of malaria transmission, but where *Anopheles superpictus* and *Anopheles stephensi* are still present as possible vectors; (iii) western part of the country with low malaria occurrence accounting for about 1–3 cases by 1000 inhabitants/year, where *An. sergentii* and *An. superpictus* are present; and, (iv) southern and southwestern parts of the country spread over the coastal plain along the Red Sea down to the border with Yemen and Tihama foothills with medium to high malaria incidence rates, where *An. sergentii* and *Anopheles arabiensis* are the main vectors [7, 8].

Even if malaria incidence is currently low, a large number of imported cases sustain potential for indigenous transmission [9]. There are three main sources of imported malaria in Saudi Arabia. The most important may be the huge number of pilgrims from around the world who visit Islamic holy places located in Saudi Arabia each year [10]. Also, the economic situation of the country is a magnet for workers in the surrounding malaria-endemic countries [11, 12] and, thirdly, the shared southern border with the Republic of Yemen, which is a malaria hyperendemic country with more than 50,000 cases notified in 2016 [1].

Controlling imported malaria becomes more crucial, especially as autochthonous endemicity has declined or even been eliminated in some areas [13], while the proportion of imported cases has expanded drastically. Imported malaria cases were accountable for 11.4% in 1998, and rose to 97.5 and 95% of malaria cases in 2011 and 2016, respectively [1, 9]. In a retrospective study, malaria was responsible for 3% of infectious disease admission to hospital in the western region of Saudi Arabia over a 5-year period [14]. Therefore, there is a clear risk that human movement can re-introduce malaria into areas where elimination has been established, resulting in the emergence of outbreaks in Saudi Arabia [15].

For a long time, chloroquine was the first-line treatment of uncomplicated malaria in the southwest of Saudi Arabia. Nevertheless, the spread of chloroquine resistance has prompted the use of sulfadoxine-pyrimethamine (SP) treatment [16]. Lately, the health authority in Saudi Arabia has embraced artemisinin-based combination therapy (ACT), comprising artesunate in addition to SP for uncomplicated *P. falciparum* infection. In severe *P. falciparum* infections, artesunate, artemether and quinine are the treatments of choice. In cases of *P. vivax*, chloroquine plus 14 days of primaquine is the treatment of choice [1].

Due to the rapid expansion of resistance to anti-malaria drugs and the special circumstance of Saudi Arabia with millions of visitors from endemic areas, including Southeast Asia where resistance to artemisinins has been characterized, it is crucial to investigate the pattern of resistance genes that are present in indigenous and imported malaria cases in the different areas of Saudi Arabia, as control strategies based on these data will most definitely diminish the prevalence of drug resistance parasites and thus improve therapeutic management of malaria cases [17] and help to achieve malaria elimination in Saudi Arabia.

The aim of this study is to analyse malaria cases characterized in a reference hospital in Taif, a city close to Mecca in the west of Saudi Arabia with a low transmission malaria rate, and to identify the genes associated with resistance that are present in the region.

## Methods

### Patients and samples

Blood samples were collected from patients coming to King Faisal hospital, Taif, Kingdom of Saudi Arabia (KSA) during the period of 6 months from February to August 2016, suffering from symptoms suggesting malaria infection (episodes of fever, chills, sweating followed by episodes of normality) and/or symptoms and signs related to blood haemolysis. Patient consent was obtained prior to collecting blood samples. Ethical approval was obtained from Taif University Research Ethics Committee (number 38-36-0019).

Giemsa-stained thin and thick blood films were prepared and rapid diagnostic test (RDT) was performed to confirm the diagnosis [18]. Blood spots were collected at the same time for DNA extraction on Whatman’s filter paper and each paper was separately saved in a sealed zip-lock plastic bag to avoid subsequent contamination. The personal data of each patient were registered including age, gender and nationality.

### Molecular identification and genotyping

DNA extraction from blood spots was carried out using the QIAamp^®^ DNA Mini Kit (QIAGEN, Germany). Two 5-sq mm blood spots were incubated at 56 °C overnight previous to DNA extraction following the manufacturer’s instructions for DNA purification from tissues. Final elution volume was in 100 µl of distilled water and stored at 4 °C until use. Malaria diagnoses were validated with a modification of the nested multiplex malaria PCR (NM-PCR) based on the small sub-unit (SSU) rRNA genes [19, 20]. The method is able to identify the 4 human malaria species (*P. vivax, P. falciparum, Plasmodium ovale*, *Plasmodium malariae*) in 2 consecutive multiplexing amplifications.

### *Plasmodium falciparum* drug resistance gene screening

Samples that were diagnosed as *P. falciparum* were further subjected to series of PCR amplification reactions targeting different genes related with drug resistance in *P. falciparum* (*pfdhfr, pfdhps, pfmdr1, pfcrt, pfcytb*, *pfketch13*) according to their authors [21–24].

Amplified gene products were subsequently subjected to sequencing to assess the occurrence of significant mutations. The amplified products were purified using Illustra DNA and gel band purification kit (General Electric Healthcare) and sequenced with the Big Dye Terminator v3.1 cycle sequencing in an ABI PRISM^®^3700 DNA analyzer. All amplified products were sequenced in both directions twice.

## Results

From all the suspected cases that were screened, 26 cases were diagnosed positive for malaria by microscopy and RDT. Multiplex PCR confirmed the diagnosis of these samples with 13 cases diagnosed as *P. falciparum* and 13 cases diagnosed as *P. vivax*. All the patients were males. The mean age of the patients was 28.1 ± SD 8.15 (24–32) years old. Different nationalities were represented among the patients of the study: Saudi, Sudanese, Ethiopian, Pakistani, Indian, and Nigerian (Table 1). Sudanese people represented 38.2% of the patients and had the highest number of *P. falciparum* (6 cases). Pakistani only presented infection by *P. vivax*, while 1 patient from Nigeria and 4 Saudi were infected by *P. falciparum*. The rest of nationalities had cases with both infections, but no mixed infections were characterized by microscopy or PCR. The 4 Saudi patients denied travelling abroad to malaria-endemic countries and they were considered as indigenous cases (Table 1). All positive *P. falciparum* samples showed wild type alleles for *pfcytb* and *pfketch13* genes. The *pfmdr*-*1* gene showed the N86Y mutation in 3 samples (23%) while no sample showed the mutation in the position 1246 of the gene (D1246Y). In 4 of the patients (31%), the triple mutation M74I, N75E, K76T for the *pfcrt* gene was found.

**Table 1:** Nationality of malaria positive cases and species involved in the infection

Nationality	*P. vivax*	*P. falciparum*	Total
Sudanese	4	6	10
Pakistani	5	0	5
Indian	3	1	4
Saudi	0	4	4
Ethiopian	1	1	2
Nigerian	0	1	1
Total	13	13	26

Mutations in the *pfdhfr* gene were present in all samples, the triple mutation (N51I, C59R, S108N), which is related to SP resistance in vitro and in vivo, were detected in 9 samples (69%) and the double mutations (N51I, S108N) were detected in 3 samples (23%). For the *pfdhps* gene almost all the samples contained one or more of the recorded gene mutations (Table 2).

**Table 2:** Mutations characterized in the different genes related to resistance to malaria drugs analyzed

Sample number	Nationality	DHPS genotype	DHFR genotype	CytB genotype	MDR genotype		CRT genotype	K13 genotype
					N86Y	D1246Y		
1	Nigerian	S436A	N51I, C59R, S108N	Wild	Wild	Wild	Wild	Wild
2	Sudanese	S436A, K540E	N51I, C59R, S108N	Wild	Wild	Wild	M74I, N75E, K76T	Wild
3	Saudi	S436A	N51I, C59R, S108N	Wild	Wild	Wild	Wild	Wild
6	Ethiopian	K540E	N51I, C59R, S108N	Wild	Wild	Wild	Wild	Wild
10	Saudi	K540E	N51I, S108N	Wild	N86Y	Wild	Wild	Wild
15	Sudanese	S436A	N51I, C59R, S108N	Wild	Wild	Wild	Wild	Wild
16	Sudanese	S436A	N51I, C59R, S108N	Wild	Wild	Wild	Wild	Wild
17	Saudi	K540E, A581G	N51I, C59R, S108N	Wild	Wild	Wild	Wild	Wild
18	Sudanese	NA	NA	NA	NA	Wild	Wild	NA
19	Sudanese	S436A	N51I, C59R, S108N	Wild	Wild	Wild	M74I, N75E, K76T	Wild
20	Indian	S436A, K540E	N51I, S108N	Wild	N86Y	Wild	M74I, N75E, K76T	Wild
21	Sudanese	A437G	N51I, S108N	Wild	Wild	Wild	Wild	Wild
26	Saudi	–	N51I, C59R, S108N	Wild	N86Y	Wild	M74I, N75E, K76T	Wild

*NA* no amplification was obtained after several trials

Regarding nationalities, *pfdhfr* and *pfdhps* are distributed in all countries, *pfmdr*-*1* N86Y was present in 2 out of 4 Saudi cases (50%) and in the Indian patient, and *pfcrt* triple mutation was found in 2 out 6 patients from Sudan (33,3%), in the Indian patient and in 1 out of 4 Saudi patients (25%). The Indian patient and one of the Saudi cases presented mutations in all genes except for *pfcytb* and *pfketch13*.

## Discussion

The first step for malaria elimination is a correct diagnosis. The microscopic observation of thick blood film, as malaria diagnosis gold standard, is a good choice for diagnosis but it is a method sometimes very subjective and very dependent of qualification of the observer. On the other hand, RDTs are not specific and in general cannot differentiate between *P. falciparum* and mixed infections. PCR, however, avoids subjectivity and is highly specific. In this case, all PCR results were confirmed by microscopy, but RDT is not useful in a context where *P. falciparum* and *P. vivax* are present in equal proportions (unlike in the main areas in Africa where *P. falciparum* is clearly dominant).

One of the most important problems in malaria control is *P. falciparum* drug resistance due to the growing resistance to almost all anti-malarial drugs, including chloroquine, amodiaquine, mefloquine, SP, artemether–lumefantrine, and lately, artemisinin [25]. After a country decides to withdraw a specific drug as a consequence of the emergence of drug resistance, the resistant parasites augment in comparison to the sensitive ones. Moreover, when countries do not take that decision as soon as drug resistance is confirmed, the mutations become permanent in the population and the possibility of using this drug as a treatment or as a prophylaxis is excluded [25]. As the KSA is actively seeking to eliminate malaria [26], molecular markers that can detect anti-malarial drug resistance represent one of the most important methods in screening for anti-malarial drugs. Markers can forecast the resistance and effectiveness to anti-malarial drugs, and point out emerging resistance in a specific area [27].

As one of the vibrant cities of the Kingdom and closely adjacent to the holy city of Mecca, where millions of pilgrims arrive every year, all year round, Taif represents a strong contender to be a focus for malaria in the Kingdom. King Faisal Hospital in Taif received 26 confirmed malaria cases over the period of 6 months (February to August 2016) of which 50% were diagnosed and then confirmed as *P. falciparum* infection. Although it is usually believed that the majority of malaria cases encountered now in the Kingdom are imported [28], 4 cases were Saudi and all of them were diagnosed as *P. falciparum*. This is an alarming reminder for the health authorities that malaria cases should not only be suspected in travellers or immigrants.

This study aimed to shed a light on probable emerging anti-malarial drug resistance in this area through the discovery of different mutations of significant genes. As the *pfcytb* and *pfketch13* genes in all of the samples were wild type, this suggests that using atovaquone or artemisinin as anti-malarial drug harbours no risk of resistance in the Taif area for now. On the other hand, the triple mutation in the *pfcrt* gene was observed in the 25% of the samples with the K76T mutation, which is the key observation in chloroquine-resistant haplotypes. Numerous studies have identified the K76T mutation as the central factor in chloroquine resistance development [25, 29–31], occurring through the replacement of lysine amino acid (K) by threonine (T) [29]. This mutation is present in all chloroquine resistance isolates from different malaria-endemic regions and can consistently be used as a chloroquine resistance marker either alone or combined with other *pfcrt* mutations. It is believed that *P. falciparum* mostly utilizes a mutant *pfcrt* product to increase efflux of chloroquine from the parasite digestive vacuole [32].

Two samples, from India and Saudi Arabia, carried a combination of mutations in the *pfmdr1* and *pfcrt* genes (N86Y + K76T), which are related to resistance to both chloroquine and amodiaquine. The N86Y substitution has been mentioned as a chloroquine modulator [33] and it is believed to be related to parasite fitness adaptation in reaction to physiological changes from *pfcrt* point mutations [34]. KSA now follows a policy using ACT, instead of the traditional chloroquine therapy, using artesunate plus SP as first-line or artemether plus lumefantrine as second-line treatment of uncomplicated *P. falciparum* [35]. SP was substituted to chloroquine as the first-line drug against malaria in some countries, but was later suspended as a consequence of the rapid occurrence of parasite resistance, but it remains valuable for intermittent preventive treatment (IPT) during pregnancy [36].

Point mutations in the *pfdhfr* and *pfdhps* genes are the chief reasons for resistance to antifolate drugs in *P. falciparum*. Point mutations affecting *pfdhfr* bestow pyrimethamine resistance, a point mutation changing Ser108 to Asn108 (S108N) in the *pfdhfr* gene increases resistance to pyrimethamine about 100-fold [37]. All the amplified samples for this gene (100%) contained the S108N mutation. Moreover, 9 out of 12 samples (75%) exhibited the triple mutation (N51I, C59R, S108N, haplotype IRN), which is a critical marker for pyrimethamine resistance, and subsequent SP failure [25, 37]. These results confirm a recent study from the southwestern region of KSA reporting the presence of the triple mutation [38].

Changes in 5 different amino acids have been observed in *pfdhps* in laboratory reference isolates: Ser436 to Ala or Phe (S436A/F), Ala437 to Gly (A437G), Lys540 to Glu (K540E), Ala581 to Gly (A581G), and Ala613 to Ser or Thr (A613S/T). No mutations have so far been described outside of these 5 codons and they were established to associate with the increase resistance to sulfadoxine and a number of other sulfonamides [37]. In the present study, all 5 mutations were found in different combination of one, 2 and sometimes 3 different mutations in the same sample (Table 2). Accumulations of mutations in *pfdhps* gene are linked with increasingly lowered sensitivity to sulfonamides [38].

Based on the findings of this research, one can anticipate the failure of SP treatment in all these cases. But in clinical observation, only 2 cases were not responsive to first-line treatment encountered (artesunate plus SP) and were then shifted to second-line treatment (artemether plus lumefantrine). The 2 patients, number 6 and 16, had the triple mutation (N51I, C59R, S108N) in the *pfdhfr* gene, while they carried the K540E or S436A in the *pfdhps* gene (Table 2). The correlation of the parasite genotype to the effect of SP treatment in a patient is affected by many factors and the parasite genotype is merely one of them. The immune response and the nutritional status of the host, along with the rates of drug metabolism and the complexity of the parasite infection, are all crucial in establishing whether a patient will overcome the infection [37]. Moreover, none of the samples showed any K13 gene mutations related to artemisinin derivatives resistance, thus the combination therapy (SP plus artesunate) can still be effective in the other patients and they even resolve the fever and other clinical manifestations.

Unfortunately, these patients risk having some resistant parasites that might overcome the drug action, continue at a low concentration for a period and then inflect a new episode. On the other hand, other researchers concluded that in case of high-grade resistance to SP treatment, failure rates are high even in the absence of artemisinin resistance [39]. When parasites contain a completely sensitive allele of *pfdhfr*, it is believed that the patient will drastically respond to SP treatment regardless of the *pfdhps* allele present. Otherwise, when a triple mutant allele of *pfdhfr* is carried, clinical failure is to be expected, and it becomes more than ever anticipated if the quadruple-mutant allele is present. In this sense, the clinical effect established will reliant on the *pfdhfr* and *pfdhps* alleles of the parasite and the individual response of the patient [37].

It is now more than a decade since the speedy appearance of lumefantrine tolerance, subsequent to artemether and lumefantrine combination treatment, was described, and now this phenotype has been reported in many endemic areas. These parasites can survive in sub-therapeutic concentrations of lumefantrine. Studies had confirmed that these phenotypes are correlated with wild-type *pfmdr1* N86Y and wild-type *pfcrt* K76T, alleles associated with chloroquine sensitivity [40]; this inverse relationship between lumefantrine and chloroquine susceptibility clarifies why lumefantrine is noted to be more efficient in parts of the world with chloroquine resistance. These lumefantrine-tolerant parasites would form the background for emergence of lumefantrine resistance [40]. Accordingly, the possibility of emergence of lumefantrine in the area of the study should be always cautiously anticipated as the percentage of *pfcrt* and *pfmdr1* wild-type genes in the samples were by far more than the mutant ones.

Although it is not conventional to change chemotherapeutic recommendations depending on molecular studies alone, it is hoped that the recognition of early markers of resistance will enhance more extensive employment of reasonable treatment policies that will delay the appearance of drug resistance against different anti-malarial drugs.

## Conclusion

The results show that haplotypes of *P. falciparum* in the western region of Saudi Arabia are exhibiting high resistance to SP. This finding would support making the decision to modify the first-line of treatment with other anti-malarial drugs, but this should be addressed after repeating the same study on a larger scale to reinforce the conclusion of the present study.

## Abbreviations

ACT: artemisinin-based combination therapy
IPT: intermittent preventive treatment
NM-PCR: nested multiplex malaria PCR
*pfdhfr*: dihydrofolate reductase
*pfdhps*: dihydropteroate synthase
*pfketch13*: kelch-propeller domains
*pfmdr1*: multidrug resistance 1 gene
*pfcrt*: chloroquine resistance transporter
*pfcytb*: cytochrome-b
RDT: rapid diagnostic test
SP: sulfadoxine-pyrimethamine
