# Correction to: Imported and autochthonous malaria in West Saudi Arabia: results from a reference hospital

**DOI:** 10.1186/s12936-018-2484-1

**Published:** 2018-09-20

**Authors:** Rasha Hassan Soliman, Patricia Garcia-Aranda, Sherine Mohamed Elzagawy, Boshra El-Sayed Hussein, Wael Wahid Mayah, Alexandra Martin Ramirez, Thuy-Huong Ta-Tang, José Miguel Rubio

**Affiliations:** 1 Microbiology Department, Faculty of Medicine, Taif University, Al Hawiyah, Taif, Kingdom of Saudi Arabia; 2 Parasitology Department, Faculty of Medicine, Suez Canal University, Ismailia, Egypt; 3 Malaria and Emerging Parasitic Diseases Laboratory, National Microbiology Centre, Instituto de Salud Carlos III, Madrid, Spain; 4 Princes Nourah Bint Abdulrahman University, Riyadh, Kingdom of Saudi Arabia; 5 Department of Tropical Medicine and Infectious Diseases, Faculty of Medicine, Tanta University, Tanta, Egypt; 6 King Faisal Medical Complex, Taif, Kingdom of Saudi Arabia; 7 Faculty of Dentisary, King Abdulaziz University, Jeddah, Kingdom of Saudi Arabia

## Correction to: Malar J (2018) 17:286 10.1186/s12936-018-2438-7

Following publication of the original article [1], it was flagged by one of the authors that the name of the *P. falciparum* gene marker of artemisinin resistance ‘pfkelch13’ was (incorrectly) written as “pfketch13”, which was repeated seven times in different parts of the published paper.

As such, please note that “pfketch13” in the article [1] should in fact be **pfkelch13**.
